# Case Report: A novel ELMOD3-ALK and EML4-ALK double-fusion responses to neoadjuvant alectinib in a lung adenocarcinoma patient

**DOI:** 10.3389/fphar.2025.1515826

**Published:** 2025-04-14

**Authors:** Kaili Huang, Wen Li, Yinyin Xue, Lei Peng, Qiang Wu, Qinghua Zhou

**Affiliations:** ^1^ Lung Cancer Center and Institute, West China Hospital, Sichuan University, Chengdu, Sichuan, China; ^2^ Department of Thoracic Surgery, West China Hospital, Sichuan University, Chengdu, Sichuan, China; ^3^ Department of Medical Oncology, Cancer Center, West China Hospital, Sichuan University, Chengdu, Sichuan, China; ^4^ Department of Radiation Oncology, Cancer Center, West China Hospital, Sichuan University, Chengdu, Sichuan, China

**Keywords:** lung adenocarcinoma, ALK, ELMOD3, double fusion, neoadjuvant alectinib

## Abstract

**Background:**

Anaplastic lymphoma kinase (ALK) rearrangements account for approximately 3%–5% of non-small cell lung cancer (NSCLC), and the echinoderm microtubule-associated protein-like 4 gene (EML4) and ALK fusion (EML4-ALK) is the most common ALK rearrangement in NSCLC patients. However, double-ALK fusion is extremely rare in clinical practice. Herein, we first report a lung adenocarcinoma patient with the coexistence of a novel subfamily 3 of ELMOD (ELMOD3)-ALK, EML4-ALK double fusion that is sensitive to alectinib target therapy.

**Materials and methods:**

Hematoxylin–eosin (H&E) staining, immunohistochemistry (IHC), and next-generation sequencing (NGS) were performed on biopsy samples.

**Results:**

The IHC analysis confirmed positive expression of ALK protein. NGS revealed a novel ELMOD3-ALK and EML4-ALK double fusion. The patient was sensitive to alectinib as neoadjuvant therapy and achieved a major pathological response (MPR), which was confirmed by the postoperative pathology diagnosis. To date, the patients’ disease-free survival (DFS) has exceeded 4 years without any significant symptoms of toxicity.

**Conclusion:**

This is the first report of one lung adenocarcinoma patient with a novel ELMOD3-ALK, EML4-ALK double-ALK fusion. This double-ALK fusion variant is sensitive to alectinib, suggesting patients with an ELMOD3-ALK, EML4-ALK double-ALK fusion could achieve clinical survival benefit from alectinib.

## Introduction

Lung cancer is a leading cause of death worldwide, and non-small cell lung cancer (NSCLC) is the most common pathological type of lung cancer (Siegel et al., 2022). The anaplastic lymphoma kinase (ALK) gene fusion accounts for about 3%–6% of NSCLC, which is the second most commonly identified targeted driver gene after epidermal growth factor receptor (EGFR) mutations in NSCLC (Yu et al., 2016). It usually occurs in light or non-smokers with lung adenocarcinoma, and the most frequent fusion is the echinoderm microtubule-associated protein-like 4 gene (EML4)-ALK variant (Koivunen et al., 2008). However, with the development and application of next-generation sequencing (NGS) technology, more than 90 uncommon fusion partners of the ALK gene have been identified in NSCLC (Ou et al., 2020). Currently, according to the National Comprehensive Cancer Network (NCCN) guidelines, the second-generation ALK-tyrosine kinase inhibitors (TKIs), such as alectinib and brigatinib, and the third-generation ALK-TKI, lorlatinib, are the preferred first-line treatment recommendations for patients with advanced NSCLC harboring ALK gene rearrangement, as they could achieve outstanding survival benefit from ALK inhibitor (ALKi) therapy (Ettinger et al., 2022). In clinical practice, double-ALK fusion remains extremely rare, and there are only a few studies reporting this phenomenon (Liang et al., 2021; Liu et al., 2024; Lin et al., 2018). Questions have arisen regarding whether these double-ALK fusion variants are heterogeneous in function, development, and treatment, and the standard of care is still unclear. In this study, we describe a patient with a novel subfamily 3 of ELMO domain-containing protein (ELMOD3)-ALK and EML4-ALK double fusion who was sensitive to alectinib.

## Case report

**FIGURE 1:**
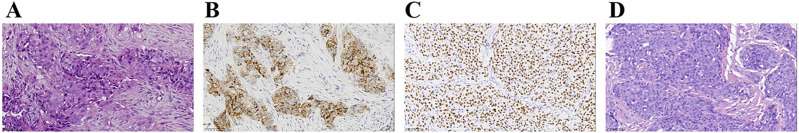
Summary of pathology results. **(A–C)** Diagnosis of lung adenocarcinoma at the time of diagnosis (40×). Hematoxylin–eosin staining of lung adenocarcinoma **(A)**. The IHC staining indicated positive for Napsin A **(B)** and TTF-1 **(C)**. **(D)** Post-operative hematoxylin–eosin staining (40×). IHC, immunohistochemistry; TTF-1, thyroid transcription factor-1.

**FIGURE 2:**
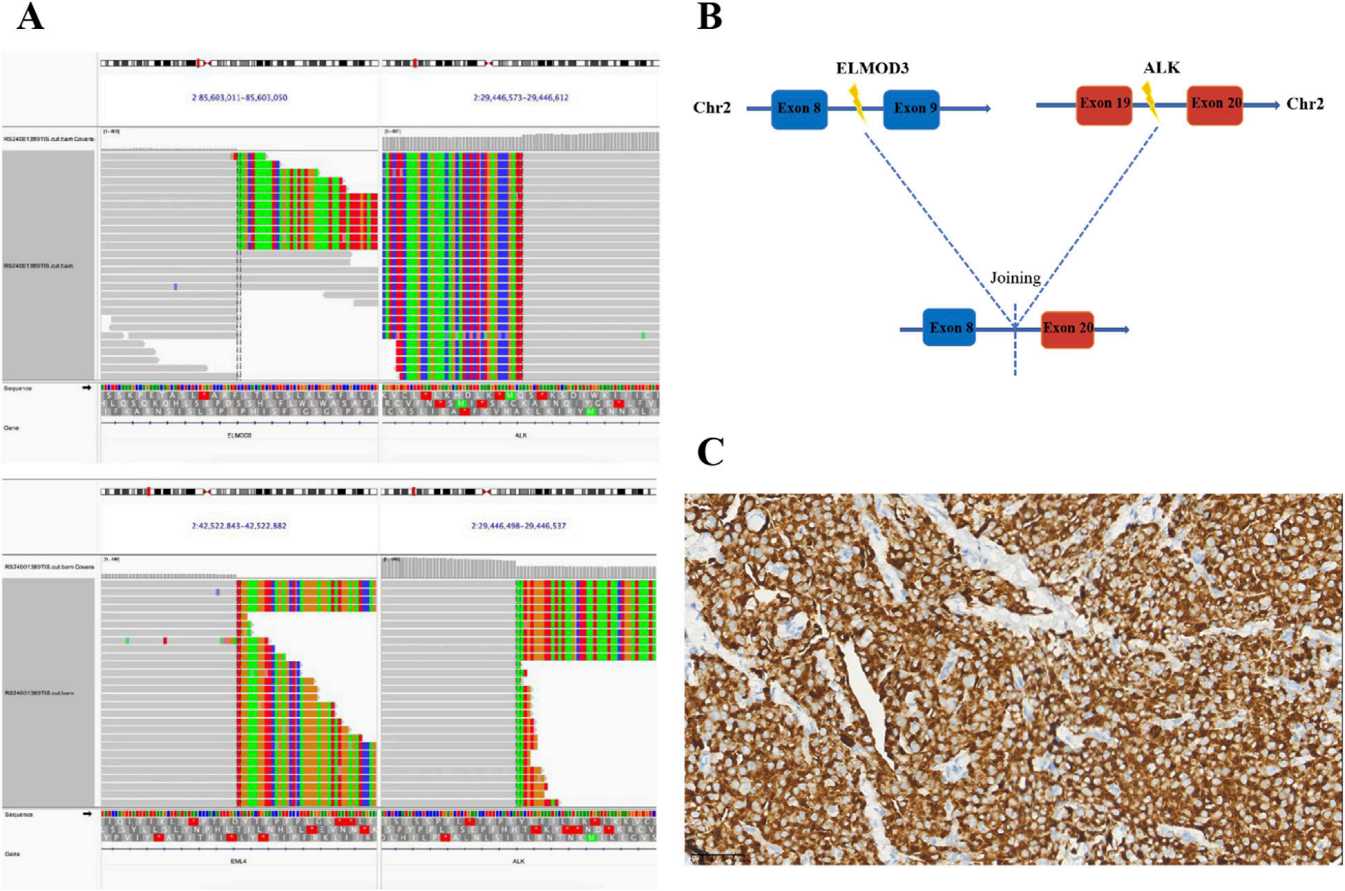
Identification of ALK fusions by NGS. **(A)** The Integrative Genomics Viewer (IVG) snapshot of a novel ELMOD3-ALK and EML4-ALK double fusion. **(B)** Illustration of the ELMOD3-ALK fusion (the new variant consists of ELMOD3 exon 8 and ALK exon 20); **(C)** IHC staining of ALK protein expression at the time of diagnosis (40×). NGS, next-generation sequencing; ALK, anaplastic lymphoma kinase; IHC, immunohistochemistry.

Four years ago, a 58-year-old Chinese male patient came to our hospital for a physical examination on 20 April 2020. The patient was asymptomatic, with no evidence of cough, sputum, hemoptysis, chest pain, or dyspnea. The patient had no history of diabetes, hypertension, or family history of cancer. The patient had a smoking history of over 7 years, with an average of 20–40 cigarettes per day, and had abstained from smoking for nearly 30 years. Chest contrast-enhanced computed tomography (CT) revealed a mass measuring approximately 4.0 cm × 3.4 cm in the inferior lobe of the right lung, along with swollen and fused lymph nodes in the hilum and mediastinum. A CT-guided lung biopsy was performed, and the pathological examination confirmed lung adenocarcinoma. Immunohistochemical (IHC) staining demonstrated that the tumor was positive for Napsin A and thyroid transcription factor 1 (TTF1) (Figure 1). Finally, the diagnosis of stage IIIA (T2aN2M0) lung adenocarcinoma was confirmed on 2 May 2020. Subsequently, NGS (Burning Rock, Guangzhou, China) was performed to detect the gene variant from the tumor tissue by lung puncture. The result identified the previously unreported ELMOD3-ALK (E8: A20) fusion and EML4-ALK (E13: A20) double-ALK fusion, with abundances of 41.23% and 68.21%, respectively. In the ELMOD3-ALK fusion, exon 8 of the ELMOD3 gene and exon 20 of the ALK gene were broken and rearranged (Figures 2A, B). The presence of the ALK fusion was further confirmed by IHC staining with the Ventana D5F3 clone (Figure 2C).

Currently, retrospective studies and case reports have demonstrated favorable efficacy and safety of neoadjuvant ALK-TKIs in resectable locally advanced NSCLC (Zhang et al., 2019; Hu et al., 2022; Sentana-Lledo et al., 2022). Additionally, a phase II, single-arm, multicenter prospective ALNEO study is ongoing to evaluate the efficacy and safety of alectinib as neoadjuvant therapy in surgically resectable stage III ALK-positive NSCLC (Leonetti et al., 2021).

**FIGURE 3:**
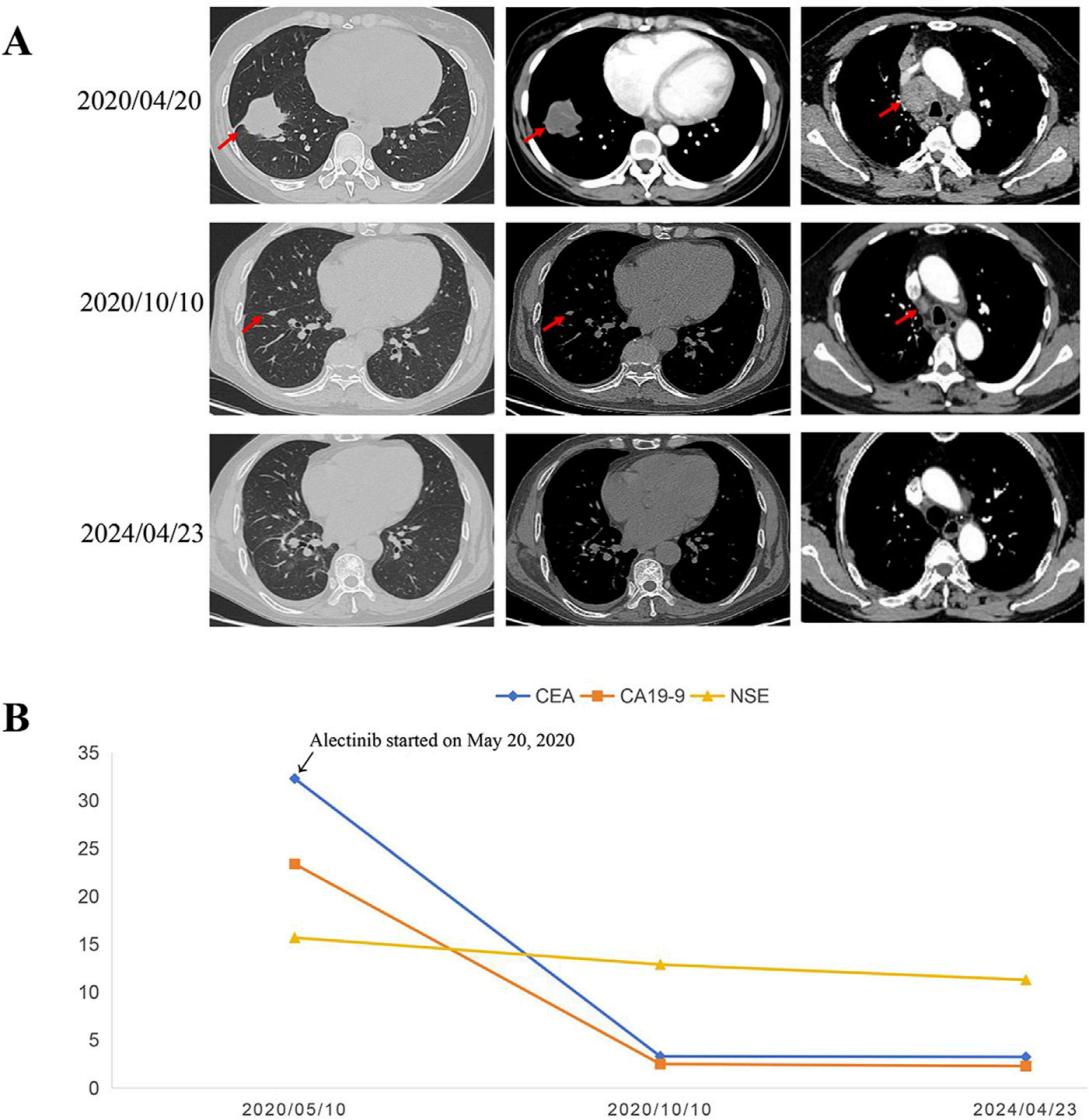
Representative CT scans and dynamic monitoring of tumor markers with timeline. **(A)** Chest CT images of right lung adenocarcinoma before and after neoadjuvant alectinib. **(B)** Dynamic monitoring of tumor markers during treatment. CT, Computed tomography.

Following a multidisciplinary team (MDT) discussion, we considered that the patient had potentially resectable NSCLC with ALK rearrangement and may benefit from the good efficacy and tolerability of ALK-TKIs. Taking into account the potential for greater side effects associated with chemotherapy and radiotherapy, as well as the possibility of increased surgical complexity due to preoperative neoadjuvant radiotherapy, we ultimately recommend neoadjuvant alectinib treatment for the patient. Thus, the patient received alectinib at a dose of 600 mg twice daily from 20 May 2020. After more than 4 months of targeted therapy of alectinib, the chest CT scan showed shrinkage of the primary tumor with a nodule measuring approximately 1.1 cm × 0.6 cm in the inferior lobe of the right lung without lymph node enlargement (Figure 3A). In addition, the CEA, NSE, and CA19-9 dropped sharply compared to initial test levels (Figure 3B). The efficacy evaluation was partial response (PR), according to response evaluation criteria in solid tumors (RECIST) 1.1.

On 15 October 2020, the patient underwent a sublobar resection of the right lower lobe and systematic mediastinal lymphadenectomy after the MDT discussion. Postoperative pathological examination revealed that the residual tumor measured approximately 1.0 cm × 0.5 cm × 0.5 cm, with the percentage of residual tumor tissue being less than 7%, achieving a major pathological response (MPR). All resected lymph nodes, including hilar and mediastinal lymph nodes, were negative for tumor cells. Additionally, NGS analysis of the residual tumor tissue detected the presence of dual ALK arrangement—ELMOD3-ALK (E8: A20) fusion and EML4-ALK (E13: A20) fusion. Therefore, the patient continued oral alectinib as adjuvant therapy postoperatively, and tumor markers, including CEA, NSE, and CA19-9, remained within normal ranges (Figure 3B). To date, the patient has continued to take alectinib (600 mg, b.i.d.) for 4 years with no recurrence of the tumor postoperatively, achieving more than 48 months of DFS without any observed serious drug-related toxicity. Follow-up is still ongoing.

## Discussion

This is the first case presenting a rare coexistence of a novel ALK double fusion, namely, ELMOD3-ALK and EML4-ALK, in a patient with lung adenocarcinoma who demonstrated favorable sensitivity to alectinib. Due to its low incidence, research on double-ALK fusions remains extremely limited. It is critical to determine the activity of different ALK fusion partners in response to different ALK-TKIs in order to make personalized and precise therapeutic decisions. This is also the first report to provide clinical evidence that NSCLC patients with ELMOD3-ALK and EML4-ALK double fusion are sensitive to alectinib and exhibit long-term benefits.

With the rapid advancements in NGS, more than 90 ALK fusion partners have been identified in NSCLC between 2007 and 2020 (Ou et al., 2020). Accumulating evidence from large-scale clinical trials demonstrates that NSCLC patients with ALK rearrangements achieve significant survival benefits from ALK-TKI therapy compared to chemotherapy (Wu et al., 2024; Novello et al., 2018). To date, the FDA has approved five ALK-TKIs for the treatment of this patient subset. However, only a limited number of studies have reported ALK double-fusion variants. In these studies, crizotinib or alectinib was used as targeted therapy, with progression-free survival (PFS) ranging from 3 to 26 months (Liang et al., 2021; Wu et al., 2020; Tao et al., 2022; Qin et al., 2019; Luo et al., 2019; Xie et al., 2024; Zeng et al., 2021). This variability suggests that different ALK fusion variants or fusion genes may result in varying levels of oncogenic ALK fusion kinase expression, leading to diverse clinical outcomes (Liang et al., 2021; Wu et al., 2020; Tao et al., 2022; Qin et al., 2019; Luo et al., 2019; Xie et al., 2024; Zeng et al., 2021), but definite evidence for this remains insufficient (Yan et al., 2020). Therefore, it is crucial for effective treatment to understand the response of different types of ALK rearrangement to ALK-TKIs.

Currently, the optimal duration of postoperative adjuvant alectinib therapy remains unclear. In the ALINA study, which focused on patients with stage IB-IIIA ALK-rearranged NSCLC, postoperative adjuvant alectinib therapy was administered for 2 years (Leonetti et al., 2021). In our case, the patient is still in a stable condition on alectinib treatment, and the DFS has already exceeded 4 years. However, the survival benefits and potential risks of longer-term adjuvant alectinib treatment are unknown and require further exploration through prospective studies. We will continue to monitor and document the patient’s subsequent treatment course.

ELMOD3 is a subfamily member of the ELMOD family, which acts on ADP ribosylation factors of regulatory GTPase (Ivanova et al., 2014). Previous studies have indicated that dysfunction of the ELMOD subfamily can lead to a variety of diseases, such as hereditary deafness, autism spectrum disorder, and intellectual disability (Jaworek et al., 2013; Loi et al., 2020). However, no studies have reported an association between the ELMOD3 gene and cancer. In this study, we report, for the first time, a novel ELMOD3-ALK and EML4-ALK double fusion identified by next-generation sequencing (NGS) in a patient with lung adenocarcinoma. This double-ALK fusion demonstrated superior clinical efficacy in response to alectinib. Therefore, we proposed that this novel ELMOD3-ALK and EML4-ALK double fusion is an alectinib-sensitive variant, and patients with this ALK double fusion may achieve survival benefit from first-line treatment of alectinib.

To our knowledge, this is the first report of a novel ELMOD3-ALK and EML4-ALK double fusion in a patient with lung adenocarcinoma. The ELMOD3-ALK (E8:A20) fusion variant has not been reported previously. Notably, neoadjuvant alectinib treatment of the patient demonstrated significant efficacy, suggesting that the ELMOD3-ALK fusion variant may be a sensitive therapeutic target. Currently, evidence regarding the efficacy and safety of alectinib as neoadjuvant therapy in locally advanced ALK-positive NSCLC remains limited. Although retrospective studies and case reports have demonstrated the benefits of neoadjuvant alectinib (Zhang et al., 2019; Hu et al., 2022; Sentana-Lledo et al., 2022), evidence from prospective clinical trials remains lacking. The ongoing ALNEO study aims to evaluate the efficacy of alectinib as neoadjuvant therapy in patients with surgically resectable stage III ALK-positive NSCLC (Leonetti et al., 2021). In the future, additional prospective clinical trials are warranted to further explore neoadjuvant targeted therapy, similar to the advancements seen in neoadjuvant immunotherapy and chemotherapy.

## Conclusion

In conclusion, we hereby report, for the first time, a case of lung adenocarcinoma harboring a novel ELMOD3-ALK and EML4-ALK double-ALK fusion. Importantly, this double-ALK fusion variant demonstrates sensitivity to first-line alectinib therapy, and patients may achieve clinical survival benefits from alectinib treatment. To further validate these findings, additional studies are expected to explore the specific molecular mechanisms underlying this double-ALK fusion and to evaluate its clinical treatment response.

## Data Availability

The original contributions presented in the study are included in the article/supplementary material; further inquiries can be directed to the corresponding authors.

## References

[B1] Ettinger D. S. Wood D. E. Aisner D. L. Akerley W. Bauman J. R. Bharat A. (2022). Non-small cell lung cancer, version 3.2022, NCCN clinical practice guidelines in oncology. J. Natl. Compr. Canc Netw. 20, 497–530. 10.6004/jnccn.2022.0025 35545176

[B2] Hu Y. Ren S. Wang R. Han W. Xiao P. Wang L. (2022). Case report: pathological complete response to neoadjuvant alectinib in a patient with resectable ALK-positive non-small cell lung cancer. Front. Pharmacol. 13, 816683. 10.3389/fphar.2022.816683 35873553 PMC9299059

[B3] Ivanova A. A. East M. P. Yi S. L. Kahn R. A. (2014). Characterization of recombinant ELMOD (cell engulfment and motility domain) proteins as GTPase-activating proteins (GAPs) for ARF family GTPases. J. Biol. Chem. 289, 11111–11121. 10.1074/jbc.M114.548529 24616099 PMC4036250

[B4] Jaworek T. J. Richard E. M. Ivanova A. A. Giese A. P. J. Choo D. I. Khan S. N. (2013). An alteration in ELMOD3, an Arl2 GTPase-activating protein, is associated with hearing impairment in humans. PLoS Genet. 9, e1003774. 10.1371/journal.pgen.1003774 24039609 PMC3764207

[B5] Koivunen J. P. Mermel C. Zejnullahu K. Murphy C. Lifshits E. Holmes A. J. (2008). EML4-ALK fusion gene and efficacy of an ALK kinase inhibitor in lung cancer. Clin. Cancer Res. 14, 4275–4283. 10.1158/1078-0432.CCR-08-0168 18594010 PMC3025451

[B6] Leonetti A. Minari R. Boni L. Gnetti L. Verzè M. Ventura L. (2021). Phase II, open-label, single-arm, multicenter study to assess the activity and safety of alectinib as neoadjuvant treatment in surgically resectable stage III ALK-positive NSCLC: ALNEO trial. Clin. Lung Cancer 22 (5), 473–477. 10.1016/j.cllc.2021.02.014 33762169

[B7] Liang Q. Xu H. Liu Y. Zhang W. Sun C. Hu M. (2021). Coexistence of a novel NBEA-ALK, EML4-ALK double-fusion in a lung adenocarcinoma patient and response to alectinib: a case report. Lung Cancer 162, 86–89. 10.1016/j.lungcan.2021.10.015 34763158

[B8] Lin H. Ren G. Liang X. (2018). A novel EML6-ALK FBXO11-ALK double fusion variant in lung adenocarcinoma and response to crizotinib. J. Thorac. Oncol. 13, e234–e236. 10.1016/j.jtho.2018.07.011 30368418

[B9] Liu Z. Huang K. Wu Q. Zhou Q. (2024). Coexistence of a novel intergenic (between CHST2 and SLC9A9)-ALK, TNIK-ALK double-fusion in resected lung adenocarcinoma. Asian J. Surg. 47, 1505–1507. 10.1016/j.asjsur.2023.11.152 38071095

[B10] Loi E. Moi L. Blois S. Bacchelli E. Vega Benedetti A. F. Cameli C. (2020). ELMOD3-SH2D6 gene fusion as a possible co-star actor in autism spectrum disorder scenario. J. Cell Mol. Med. 24, 2064–2069. 10.1111/jcmm.14733 31800155 PMC6991669

[B11] Luo J. Gu D. Lu H. Liu S. Kong J. (2019). Coexistence of a novel PRKCB-ALK, EML4-ALK double-fusion in a lung adenocarcinoma patient and response to crizotinib. J. Thorac. Oncol. 14, e266–e268. 10.1016/j.jtho.2019.07.021 31757376

[B12] Novello S. Mazières J. Oh I. J. de Castro J. Migliorino M. R. Helland Å. (2018). Alectinib versus chemotherapy in crizotinib-pretreated anaplastic lymphoma kinase (ALK)-positive non-small-cell lung cancer: results from the phase III ALUR study. Ann. Oncol. 29, 1409–1416. 10.1093/annonc/mdy121 29668860 PMC6005013

[B13] Ou S. I. Zhu V. W. Nagasaka M. (2020). Catalog of 5' fusion partners in ALK-positive NSCLC circa 2020. JTO Clin. Res. Rep. 1, 100015. 10.1016/j.jtocrr.2020.100015 34589917 PMC8474466

[B14] Qin B. D. Jiao X. D. Liu K. Wu Y. Zang Y. S. (2019). Identification of a novel EML4-ALK, bcl11a-ALK double-fusion variant in lung adenocarcinoma using next-generation sequencing and response to crizotinib. J. Thorac. Oncol. 14, e115–e117. 10.1016/j.jtho.2019.01.032 31122560

[B15] Sentana-Lledo D. Viray H. Piper-Vallillo A. J. Widick P. Rangachari D. Wilson J. L. (2022). Complete pathologic response to short-course neoadjuvant alectinib in mediastinal node positive (N2) ALK rearranged lung cancer. Lung Cancer 172, 124–126. 10.1016/j.lungcan.2022.08.014 36075183 PMC9719796

[B16] Siegel R. L. Miller K. D. Fuchs H. E. Jemal A. (2022). Cancer statistics, 2022. CA Cancer J. Clin. 72, 7–33. 10.3322/caac.21708 35020204

[B17] Tao H. Liu Z. Mu J. Gai F. Huang Z. Shi L. (2022). Concomitant novel ALK-SSH2, EML4-ALK and ARID2-ALK, EML4-ALK double-fusion variants and confer sensitivity to crizotinib in two lung adenocarcinoma patients, respectively. Diagn Pathol. 17, 27. 10.1186/s13000-022-01212-9 35144623 PMC8832643

[B18] Wu X. Wang W. Zou B. Li Y. Yang X. Liu N. (2020). Novel NLRC4-ALK and EML4-ALK double fusion mutations in a lung adenocarcinoma patient: a case report, Thorac. Cancer, 11:1695–1698. 10.1111/1759-7714.13389 32212216 PMC7262889

[B19] Wu Y. L. Dziadziuszko R. Ahn J. S. Barlesi F. Nishio M. Lee D. H. (2024). Alectinib in resected ALK-positive non-small-cell lung cancer. N. Engl. J. Med. 390, 1265–1276. 10.1056/NEJMoa2310532 38598794

[B20] Xie X. Guan W. Huang W. Jiang J. Deng H. Li Y. (2024). Coexistence of a novel SRBD1-ALK, ALK-CACNA1D double-fusion in a lung adenocarcinoma patient and response to alectinib: a case report. Heliyon 10, e24373. 10.1016/j.heliyon.2024.e24373 38312631 PMC10835179

[B21] Yan J. Zhou X. Pan D. (2020). A case of one lung adenocarcinoma patient harboring a novel FAM179A-ALK (F1, A19) rearrangement responding to lorlatinib treatment. Lung Cancer 147, 26–29. 10.1016/j.lungcan.2020.06.026 32652371

[B22] Yu Y. Ding Z. Zhu L. Teng H. Lu S. (2016). Frequencies of ALK rearrangements in lung adenocarcinoma subtypes: a study of 2299 Chinese cases. Springerplus 5, 894. 10.1186/s40064-016-2607-5 27386342 PMC4923004

[B23] Zeng Q. Gao H. Zhang L. Qin S. Gu Y. Chen Q. (2021). Coexistence of a secondary STRN-ALK, EML4-ALK double-fusion variant in a lung adenocarcinoma patient with EGFR mutation: a case report. Anticancer Drugs 32, 890–893. 10.1097/CAD.0000000000001094 34232939

[B24] Zhang C. Li S. L. Nie Q. Dong S. Shao Y. Yang X. N. (2019). Neoadjuvant crizotinib in resectable locally advanced non-small cell lung cancer with ALK rearrangement. J. Thorac. Oncol. 14 (4), 726–731. 10.1016/j.jtho.2018.10.161 30408570

